# Regulation of *Drosophila* Eye Development by the Transcription Factor Sine oculis

**DOI:** 10.1371/journal.pone.0089695

**Published:** 2014-02-25

**Authors:** Barbara Jusiak, Umesh C. Karandikar, Su-Jin Kwak, Feng Wang, Hui Wang, Rui Chen, Graeme Mardon

**Affiliations:** 1 Program in Developmental Biology, Baylor College of Medicine, Houston, Texas, United States of America; 2 Department of Pathology and Immunology, Baylor College of Medicine, Houston, Texas, United States of America; 3 Department of Molecular and Human Genetics, Baylor College of Medicine, Houston, Texas, United States of America; 4 Human Genome Sequencing Center, Baylor College of Medicine, Houston, Texas, United States of America; 5 Department of Neuroscience, Baylor College of Medicine, Houston, Texas, United States of America; 6 Department of Ophthalmology, Baylor College of Medicine, Houston, Texas, United States of America; 7 Program in Cell and Molecular Biology, Baylor College of Medicine, Houston, Texas, United States of America; Indiana University, United States of America

## Abstract

Homeodomain transcription factors of the Sine oculis (SIX) family direct multiple regulatory processes throughout the metazoans. Sine oculis (So) was first characterized in the fruit fly *Drosophila melanogaster*, where it is both necessary and sufficient for eye development, regulating cell survival, proliferation, and differentiation. Despite its key role in development, only a few direct targets of So have been described previously. In the current study, we aim to expand our knowledge of So-mediated transcriptional regulation in the developing *Drosophila* eye using ChIP-seq to map So binding regions throughout the genome. We find 7,566 So enriched regions (peaks), estimated to map to 5,952 genes. Using overlap between the So ChIP-seq peak set and genes that are differentially regulated in response to loss or gain of *so*, we identify putative direct targets of So. We find So binding enrichment in genes not previously known to be regulated by So, including genes that encode cell junction proteins and signaling pathway components. In addition, we analyze a subset of So-bound novel genes in the eye, and find eight genes that have previously uncharacterized eye phenotypes and may be novel direct targets of So. Our study presents a greatly expanded list of candidate So targets and serves as basis for future studies of So-mediated gene regulation in the eye.

## Introduction

The homeodomain transcription factor Sine oculis (So) is a member of the highly conserved Retinal Determination (RD) gene network, which consists of transcriptional regulators that are both necessary and sufficient for eye development in *Drosophila* (reviewed by [1]). So regulates multiple aspects of eye development and is expressed in the larval precursor structure to the adult eye – the eye imaginal disc. So expression begins in the eye imaginal disc during the second instar larval stage, when the eye disc consists of proliferating retinal progenitor cells. At the beginning of the third instar stage, an indentation called the morphogenetic furrow (MF) forms at the posterior margin of the eye disc, marking the onset of cell differentiation, and sweeps progressively across the eye disc toward the anterior margin [2]. So is expressed in a narrow domain anterior to the MF and in all the cells posterior to the MF including the differentiating cells [3], [4]. In the eye specific mutant *so^1^*, So is not expressed in the eye discs. The absence of So expression blocks MF initiation as well as retinal differentiation leading to massive apoptosis of the retinal progenitor cells and adult flies without eyes [3]. Consistent with these observations, clonal analysis using *so* null mutant alleles indicates that *so* is required for MF initiation and progression, as well as the differentiation or survival of photoreceptor precursors posterior to the MF [5]. Despite the vital role of So in eye development, only a few direct So targets have been identified to date. Most of these targets encode transcriptional regulators necessary for various stages of eye development, including the RD network genes *eyeless* (*ey*) and *dachshund* (*dac*), as well as genes that direct retinal differentiation such as *atonal* (*ato*), *lozenge* (*lz*), and *prospero* (*pros*) [6], [7], [8], [9], [10]. So also regulates the expression of *hedgehog* (*hh*), which encodes a secreted ligand that drives MF progression in the eye disc [8].

In order to improve our understanding of how So regulates eye development, we have sought to identify targets of So during eye development on a genome-wide scale. To this end we have performed chromatin immunoprecipitation with an anti-So antibody followed by genome-wide sequencing (ChIP-seq) on third instar eye-antennal discs. We found 7,566 regions were enriched for So binding throughout the genome. These So-bound regions (referred to as peaks) correspond to estimated 5,952 genes. The So-enriched genes include previously characterized direct targets of So, indicating that ChIP-seq can identify biologically relevant targets of So. As expected, the genes enriched for highly significant So peaks are over-represented in Gene Ontology categories that pertain to eye development. In addition, many So peaks map to genes that have not been known to function during eye development. We have obtained mutant alleles of a subset of these So-enriched, novel genes, and have assayed their function in the eye. We have identified eight novel genes that are necessary for eye development and may act downstream of So. In addition, we have intersected our ChIP-seq data set with genome-wide changes in expression profiles due to loss or gain of *so* to identify candidate genes that are directly regulated by So in the eye. Together, our results greatly expand the set of putative So targets in the developing eye, and set the stage for many future studies of So function in development.

## Materials and Methods

### Chromatin immunoprecipitation

Chromatin immunoprecipitation protocol was performed as previously described [11]. 400 eye-antennal disc complexes (including mouth hooks, but not brains) from *white^1118^* wandering third instar larvae were used per replicate; two biological replicates were conducted. The chromatin sample was incubated with 1∶500 guinea pig anti-So antibody (kind gift from Ilaria Rebay; [12]), and an identical chromatin sample incubated without antibody served as negative control. ChIP-seq libraries were prepared according to the Illumina ChIP-seq library protocol. High-throughput library sequencing was performed using the Illumina Genome Analyzer IIx. The 35 bp reads from the two biological replicates were combined and then mapped to the *Drosophila* genome using Eland software.

### Peak mapping and analysis

The reads were mapped and peaks were called using Model-based Analysis of ChIP-seq (MACS; http://liulab.dfci.harvard.edu/MACS/) [13]. Peaks with a P value of larger than 10^−5^ or with a fold change of less than 3 were filtered out. Gene Ontology analysis of genes with the top 10% most highly enriched peaks was performed using the publicly available Database for Annotation, Visualization and Integrated Discovery (DAVID; http://david.abcc.ncifcrf.gov) [14]. *De novo* motif analysis of the entire ChIP-seq peak set was carried out with the publicly available Regulatory Sequence Analysis Tool (RSAT) software (http://rsat.ulb.ac.be/rsat/) [15]. So ChIP-seq data have been deposited in NCBI Gene Expression Omnibus with the accession number GSE52943 (http://www.ncbi.nlm.nih.gov/geo/query/acc.cgi?acc=GSE52943).

### Screen for novel putative targets of So with eye phenotypes

We ordered transposon insertions and mutant alleles of previously uncharacterized genes enriched for So binding, if available, from the Bloomington *Drosophila* Stock Center (BDSC) at Indiana University, Bloomington (http://flystocks.bio.indiana.edu); the *Drosophila* Genetic Resource Center (DGRC) at Kyoto Institute of Technology, Japan (http://www.dgrc.kit.ac.jp/en/index.html); and the Carnegie collection [16]. Homozygous lethal lines were subjected to a complementation test with a molecularly mapped deficiency ordered from BDSC that uncovers the gene of interest (*goi*). If a mutant failed to complement the deficiency, indicating that the lethality mapped to the region of interest, we proceeded to recombine the mutant allele onto a suitable FRT chromosome: *FRT19A* for X, *FRT40A* for 2L, *FRT24D* for 2R, *FRT80B* for 3L, and *FRT82B* for 3R. In case of *w^+^* transposon lines, putative recombinants were identified by eye color (*w^+^* animals that survived on G418), crossed individually to suitable balancer flies (*FM7c/Y* for the X chromosome, *w; BcE/CyO* for the 2^nd^ chromosome, and *w; TM3/TM6B, Tb* for the 3^rd^), and then verified by single-fly genomic PCR using pairs of primers spanning the two transposon/genomic region junctions. For *w^−^* transposon lines and point mutants, individual flies were tested by PCR (for transposon lines) or failure to complement the deficiency corresponding to the gene of interest.

The recombinant *w; FRT goi/Bal* males were crossed with *ey-FLP; FRT cl/Bal* virgins in order to test the requirement of the mutant gene in eye development, yielding progeny of the genotype *ey-FLP/(w or Y); FRT goi/FRT cl*. *ey-FLP* drives expression of Flippase (Flp) in the early eye disc, leading to *FRT* recombination and the formation of homozygous *goi* and *cl* clones [17]. The *cl* homozygous cells die due to the recessive cell lethal (*cl*) allele, leaving the eye disc composed mainly of homozygous *goi* cells. We scored the external eye phenotype of *ey-FLP/w; FRT goi/FRT cl* flies. If we saw defects, such as reduced or rough eye, we performed adult eye sections as previously described [18]. Table 1 lists stocks that were found to cause adult eye defects.

**Table 1:** Mutant alleles associated with novel eye phenotypes.

Gene	Allele	Nature of allele	Eye color^a^	Reference
*blot*	*01658*	*P*-element in intron near 5′UTR	*w^−^*	[82]
*CG12007*	*LL03248*	*Piggybac* in ORF	Strong *w^+^*	[83]
*CG13192*	*EY07746*	*P*-element in ORF	Weak *w^+^*	FBrf0132177^b^
*CG2747*	*KG09552*	*P*-element in 5′UTR	Strong *w^+^*	FBrf0132177^b^
*CG8108*	*EY14316*	*P*-element in ORF	Weak *w^+^*	FBrf0132177^b^
*l(3)j2D3*	*j2D3*	*P*-element in ORF	Strong *w^+^*	[84]
*omd*	*EY04837*	*P*-element in 5′UTR	Weak *w^+^*	FBrf0132177^b^
*Syp*	*03806*	*P*-element in intron^c^	*w^−^*	[84]

a Eye color of homozygous mutant tissue in the adult. Weak *w^+^* and *w^−^* tissue can be distinguished from heterozygous tissue by color; strong *w^+^* tissue cannot.

b Personal communication to FlyBase; related publication: [85]

c The allele is in the 5′UTR of *CG31195,* which maps to an intron of *Syp,* and hence the phenotype may potentially be due to loss of expression of *CG31195.*

A few transposon insertions of interest had been recombined onto an *FRT* chromosome previously by the UCLA Undergraduate Research Consortium in Functional Genomics (URCFG; http://www.bruinfly.ucla.edu/). Where possible, we ordered these flies, performed complementation tests, and proceeded directly to assaying the eye phenotype with *ey-FLP; FRT cl/Bal.*

## Results

### So ChIP-seq binding profile

Given the importance of *so* in *Drosophila* eye development, we used the genome-wide ChIP-seq approach to identify genes that may be directly regulated by So. Mid-third instar eye discs were chosen for the analysis as they exhibit retinal progenitors in different stages of eye development. Thus, performing So ChIP-seq at this stage is expected to identify targets of So in retinal progenitors ahead of the MF as well as in cells undergoing differentiation posterior to the MF. We performed two biological replicates of So ChIP-seq that yielded ∼4.74 million 35 bp reads, of which ∼3.4 million reads are unique. The combined unique reads from the two biological replicates were mapped to the *Drosophila melanogaster* FlyBase genome release 05, resulting in 7,566 regions enriched for So binding. These So-bound regions are henceforth referred to as ‘So-ChIP-seq peaks’. All ChIP-seq peaks are listed in Dataset S1.

**Figure 1:**
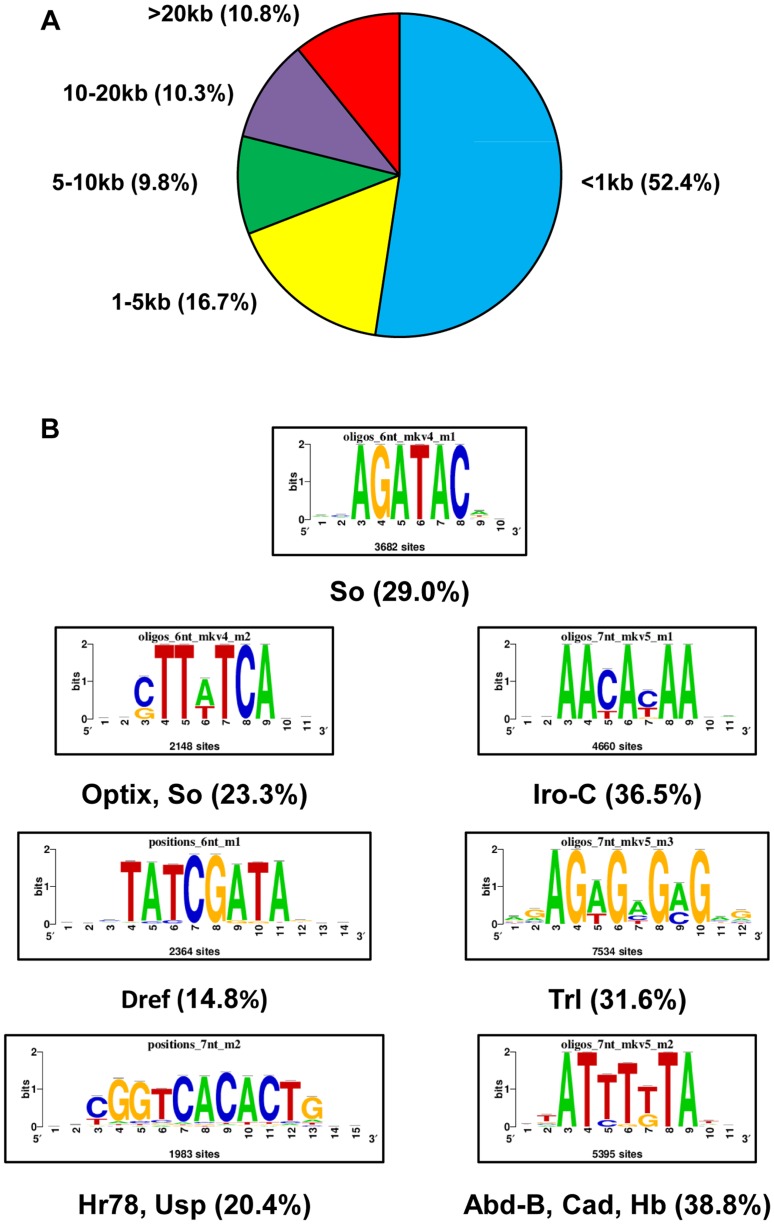
Peak distribution and motif enrichment of the So ChIP-seq data set. (A) Genome distribution of So-enriched regions (peaks) with respect to annotated transcription start sites (TSS). 52.4% of So peaks are <1 kb from the nearest TSS, and 78.9% of peaks are no more than 10 kb from the nearest TSS. (B) Transcription factor binding motifs enriched in the So ChIP-seq peak set. The name of the TF and the percentage of peaks that have at least one occurrence of the TF motif are listed under each motif. Abd-B, Abdominal-B; Cad, Caudal; Dref, DNA Replication Element factor; Hb, Hunchback; Hr78, Hormone receptor 78; Iro-C, Iroquois Complex; Trl, Trithorax-like; Usp, Ultraspiracle.

The So-ChIP-seq peaks were associated with estimated 5,952 genes with a mean peak width of ∼1 kb. The majority of the So-ChIP-seq peaks (84.7%) overlap one or more genes at least partially, and these peaks were assigned to the gene(s) they overlap. 15.3% of So-binding peaks are intergenic peaks, which were assigned to the nearest gene in either direction. Most peaks are less than 5 kb from an annotated transcription start site (TSS), with 52.4% of the peaks being <1 kb from a TSS, and 16.7% of peaks between 1 and 5 kb from the nearest annotated TSS. Only 10.8% of the peaks are more than 20 kb from an annotated TSS (Figure 1).

### So ChIP-seq identifies previously known direct targets of So

**Figure 2:**
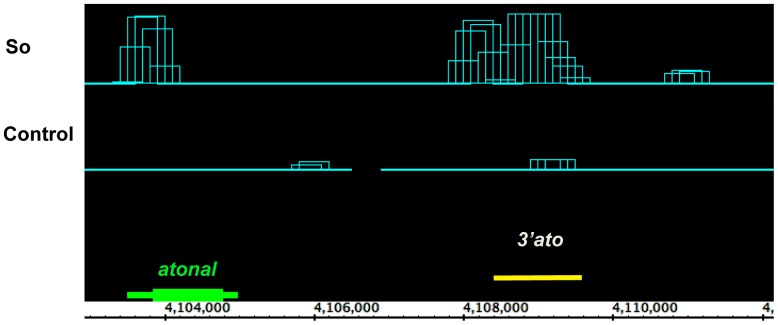
So ChIP-seq peak in the So target gene *atonal* maps to a previously known So-regulated enhancer. The top panel (“So”) shows the So binding profile, which is significantly enriched compared with the negative control (“Control” panel). The bottom panel shows the *atonal* (*ato*) protein coding region in green. The yellow bar in the bottom panel marks the *3*′ *ato* enhancer, which is necessary for the onset of *ato* in the eye and is directly activated by So [10]. The So ChIP-seq peak overlaps this previously identified *3*′ *ato* enhancer.

To date, seven direct targets of So during eye development have been identified, namely *ey, lz*, *so*, *hh*, *dac, ato*, and *pros*. Enhancer analysis for these genes has suggested that So regulates their expression during eye development [6], [7], [8], [9], [10]. We mapped the known So-dependent enhancers of the seven known So targets to our ChIP-seq data set to see how many of these So dependent enhancers are bound by So in the developing eye disc. Our So ChIP-seq analysis showed that six out of seven genes are enriched for So-binding at the published So-dependent enhancers. For example, the *ato* enhancer that harbors a So-binding site is bound by So in the third instar eye disc (Figure 2, [10]). Our data accurately identified six out of seven published So-dependent enhancers, suggesting that our ChIP-seq data can predict biologically relevant targets. Although we do observe an So peak within the *ey* gene, it does not overlap the previously reported eye enhancer [8].

### So peaks are enriched for transcription factor binding motifs

Previous studies suggest that So acts with other transcription factors to regulate the expression of its target genes. For example, So and Ey bind to adjacent motifs in an *ato* enhancer, and both are necessary for the onset of *ato* expression in the eye [10]. So also cooperates with PntP2, the downstream effector of Egfr signaling, in activating *hh* and *pros* expression during eye development [6], [19], [20]. Therefore, we tested whether the So bound regions are enriched for binding motifs of other transcriptional regulator(s) in order to identify synergistic interactions between So and other DNA binding TFs that play a role during eye development. Toward this end we used the RSAT program [15] to identify binding motifs enriched within the So ChIP-seq peaks.

Analysis of the entire So ChIP-seq peak set with RSAT software revealed enrichment for several motifs (Figure 1B). One of the top hits among these is AGATAC, which closely matches the consensus motif for So, YGATAY (Y = C/T) [11]. A second enriched motif, STTWTCA (S = C/G, W = A/T), matches the consensus motif for the So paralog and RD network member Optix, which is expressed along with So in the anterior portion of the eye disc [21]. A third enriched motif is AACAYAA, the motif for homeodomain transcription factors of the Iroquois Complex, which establish dorsal-ventral polarity in the eye disc – Mirror (Mirr), Caupolican (Caup), and Araucan (Ara) [22], [23]. A fourth enriched motif is TATCGATA, which corresponds to the DNA Replication Element (DRE), the binding site for the zinc finger protein DRE factor (Dref) [24], while a fifth motif, AGAGMGMG (M = A/C), resembles the consensus for Trl (Trithorax-like), a zinc finger chromatin remodeling protein. A sixth motif, CGGTCACACTG, corresponds to the nuclear hormone receptor Hr76 and to Ultraspiracle (Usp), which mediates the transcriptional response to the hormone ecdysone [25]. ATTTKTA (K = G/T), a seventh enriched motif, resembles the motifs for several transcription factors that are important for determining the embryonic body plan and specification of embryonic as well as larval retinal fields, including the homeodomain factors Abdominal-B (Abd-B), Caudal (Cad), and Zerknüllt (Zen); the zinc finger transcription factors Hunchback (Hb) and Broad-Z1 (Br-Z1); and the helix-loop-helix factor Bric a brac 1 (Bab1).

### So binds to or near genes predicted to function in eye development

To identify major biological processes associated with genes enriched for So binding, we performed Gene Ontology (GO) analysis. We ranked the ChIP-seq peaks based on their P-values and selected the top 10% most significant ChIP-seq peaks (757 peaks corresponding to 782 annotated protein-coding genes, P≤10^−64^). The GO analysis of genes that correspond to the top 10% most significant ChIP-seq peaks was done using the program DAVID [26], [27], to obtain enrichment scores for different GO terms. Highly enriched GO terms (enrichment score >1.3, corresponding to P<0.05) include terms predicted to be associated with putative So target genes, such as *Imaginal disc development*, *Sensory organ development*, *Neuron differentiation*, and others (Table S1).

There are also GO clusters unrelated to eye development, including *Gland development*, *Ovarian follicle cell development*, *Leg disc development*, and *Muscle attachment*, among others (Table S1). Some of these terms may be expected since some putative So targets are known to function in multiple developing organs and tissues. For example, *Leg disc development*, an enriched GO term, requires *dac*, a direct target of So in the eye [28], [29]. In addition to eye development, So/SIX transcription factors, along with other members of the RD network, orchestrate multiple developmental processes. For example, enrichment for GO terms associated with muscle may reflect the requirement for the So cofactor Eya in the developing muscle [30]. The Notch and Epidermal growth factor receptor (Egfr) signaling pathways (reviewed by [31]) are also employed in the development of multiple organs and tissues, and several components of each pathway are present among genes corresponding to the top 10% of the So peaks.

Several enriched GO terms refer to processes that are not currently known to be regulated by So during eye development. It is possible that the presence of genes associated with these developmental process may reflect a previously undescribed role for So. These GO terms include *Protein kinase cascade*, *Cell-cell junction organization*, and *Asymmetric protein localization* (Table S1). Thus GO analysis of the top putative targets of So based on So-binding suggests that our So ChIP-seq analysis contains genes involved in diverse processes during eye development including those that were not previously associated with So function.

### Identification of novel eye genes

**Figure 3:**
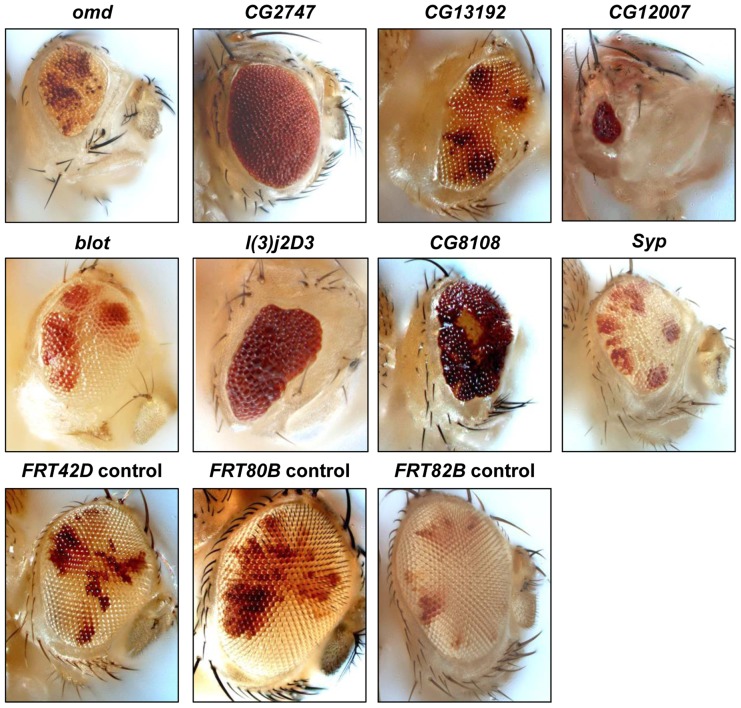
Novel eye phenotypes of putative So target genes. Adult eye phenotypes were analyzed using *ey-FLP; FRT goi/FRT cl.* Homozygous mutant tissue has less pigmentation than heterozygous tissue in *omd, CG13192, blot, CG8108,* and *Syp.* In *CG2747, CG12007,* and *l(3)j2D3,* homozygous vs. heterozygous tissue cannot be distinguished by color. *omd, CG2747, CG12007,* and *Syp* are on chromosome 3R (*FRT82B*); *blot, l(3)j2D3,* and *CG8108* are on 3L (*FRT80B*); and *CG13192* is on 2R (*FRT42D*). Bottom panel shows control external eye phenotypes of *ey-FLP; FRT/FRT cl* flies.

**Figure 4:**
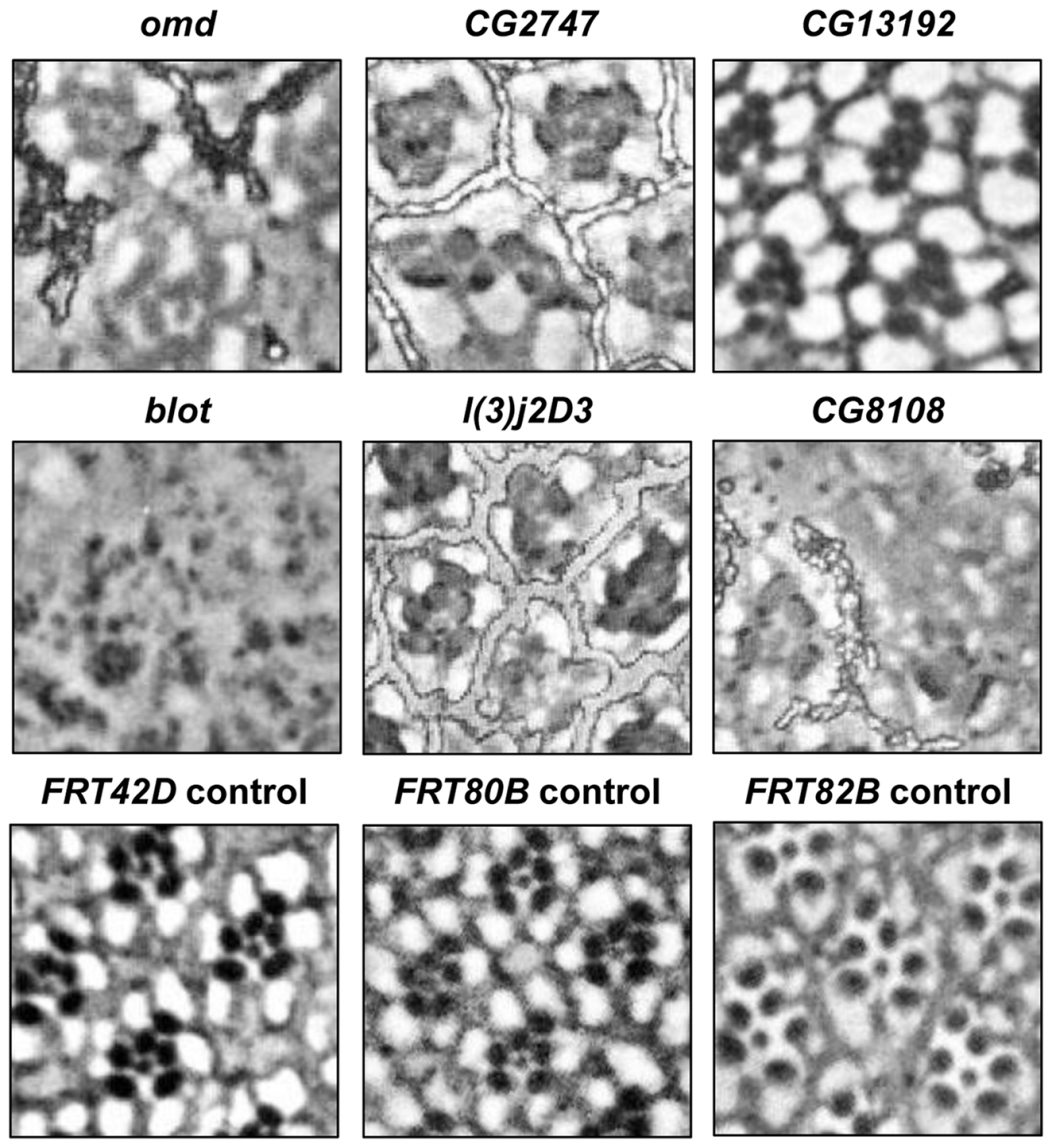
Ommatidial defects in putative So target gene mutants. Top panel: tangential sections through adult eyes of *ey-FLP; FRT goi/FRT cl* flies reveal defects ranging from loss of the inner photoreceptor (*CG13192*) to complete loss of rhabdomeres (*blot*). Bottom panel: Control sections through the eyes of *ey-FLP; FRT/FRT cl* flies.

Over half of the genes that harbor So peaks that rank among the top 10% based on P-value (≥642.39, corresponding to P≤10^−64^) have no previously described role in eye development. Some of these genes have been shown to play roles in the development of other organs such as gonad or brain, some cause early lethality, and the mutant phenotypes for many have not been reported. To expand our understanding of how So regulates eye development, we investigated if these genes with highly enriched So ChIP-seq peaks are required for eye development. To this end, we compiled a list of genes that met the following criteria: 1) the role of the gene in the eye had not been reported; 2) a So peak with P<10^−20^ maps to the gene; and 3) the gene is expressed (expression level ≥10) in wild-type third instar eye discs based on previously published microarray data [32]. We then obtained available alleles, including P-element insertions, in these genes from the *Drosophila* stock centers (see Materials and Methods). Recessive lethal alleles that failed to complement a deficiency covering the gene of interest were recombined onto an *FRT* chromosome in order to assay the phenotype in the eye using the *ey-FLP/FRT* technique [17], [20]. Out of 26 alleles tested with *ey-FLP*, loss-of-function mutations in fifteen genes had no effect on adult eye morphology (*4EHP, akirin, CG43658, CG6767, CG7675, CG8223, CG9932, cnc, Eaf6, ems, Imp, l(3)L1231, Mpcp, vkg,* and *wde*), and loss of two genes caused very subtle disorganization of the adult eye (*CG1965* and *att-ORFA*); these 17 genes were not analyzed further. Eight showed severe defects in adult eye morphology and a ninth gene, *krotzkopf verkehrt* (*kkv*/*CG2666*), resulted in pharate lethal flies with severe reduction of the head (data not shown). Eight mutant lines resulted in viable flies with reduced and misshapen eyes (Figure 3). These mutants lead to a range of defects in ommatidial architecture from minor disorganization to complete loss of ommatidia as compared to the control (Figure 4): *bloated tubules* (*blot/CG3897*), *CG12007, CG13192, CG2747, CG8108, l(3)j2D3/CG6801, oocyte maintenance defects* (*omd/CG9591*), and *Syncrip* (*Syp/CG17838*) (Table 1).

One of the novel genes, *omd*, is of particular interest due to its proposed role as a negative regulator of Decapentaplegic (Dpp) signaling [33], which is necessary for normal eye development [34]. *omd* has a So ChIP-seq peak (P = 10^−56^) that maps to the first intron and first coding exon of *omd*, as well as to the TSS of an overlapping gene, *falafel* (*flfl/CG9351*). Given the overlap between *omd* and *flfl* it is not possible to determine whether the So-ChIP-seq peak reflects So-mediated regulation of *omd*, *flfl*, or both. We have used a lethal P-element insertion in the 5′ UTR of *omd* (*omd^EY04837^*) to test if the *omd/flfl* locus plays a role during eye development (it is unknown whether the P-element lethality is due to disruption of *omd* or *flfl*). Clonal analysis using *omd^EY04837^* results in reduced and disorganized adult eyes, with some homozygous mutant tissue surviving in the adult (Figure 3 and 4). *omd* encodes a subunit of the Integrator complex, which processes small nuclear RNAs (snRNA) [35]. Knockdown of other Integrator complex subunits causes multiple developmental defects in *Drosophila*, including in the embryo, wings, and bristles [36]. In addition, an RNAi screen has identified *omd* as a putative regulator of neural stem cell self-renewal in the larval brain [37]. The *flfl* gene also has a role in neurogenesis: it encodes a protein phosphatase regulatory subunit that regulates asymmetric protein localization in dividing neuroblasts [38].

### Transcriptional profiling to detect putative So targets

So ChIP-seq detects So binding to genes in the third instar eye disc, but not necessarily the regulation of a target gene by So. If a gene is a putative target of So, we predict that its expression would be affected by changes in So levels. Therefore, we reasoned that putative direct So targets can be identified as the genes that are bound by So in the eye disc and that are differentially regulated in response to changes in *so* expression. However, observing transcriptional changes in the *so* mutant eye disc is challenging, because *so^1^* homozygous mutant eye discs undergo massive apoptosis in the early third instar stage, around the time when wild type eye discs initiate differentiation [3]. Therefore, we used an alternative approach by monitoring changes in gene expression during ectopic eye formation in *Drosophila* imaginal discs. The changes in gene expression were analyzed by microarray analysis [32]. Previous studies have shown that overexpression of transgenes encoding the RD network members Ey, So, and Eya can lead to eye formation in ectopic sites such as legs [39], [40], [41]. We have used a combination of microarray data sets analyzing the gene expression changes due to loss and gain of *so* during ectopic eye induction ([32] and our unpublished data). In the current study, we used the microarray data in two ways to identify candidate direct targets of So.

The first approach was based on the observation that the RD gene *eyeless* (*ey*) is a potent inducer of ectopic eyes in multiple imaginal discs, but it cannot induce ectopic eye formation in *so^1^* mutant discs [40], [42]. This observation suggests that similar to normal eye development, ectopic eye development requires *so*. Therefore, we compared the gene expression profiles of leg imaginal discs expressing a *UAS-ey* (*Uey*) transgene in presence and absence of endogenous *so* (wild-type and *so^1^* mutant background, respectively) to identify genes whose expression is affected by loss of *so* function.

Second, overexpression of *so* alone is a weak inducer of ectopic eyes and, consistent with this observation, few genes are differentially regulated between wild type and *so*-overexpressing leg discs ([41] and our unpublished data). However, co-overexpression of *so* and *eya*, which encodes a transcriptional coactivator that binds So, results in robust induction of ectopic eyes [5], [43]. Therefore, we compared gene expression profiles of wild-type leg discs to those that co-overexpress *so* and *eya* (*Ueya+so*) transgenes in order to identify genes that respond to elevated levels of *so* and/or *eya* (we note that this approach does not distinguish genes that respond to the So/Eya complex from genes that respond to Eya alone).

Putative direct targets of So were identified as genes that show either altered expression in *ey-*overexpressing leg discs in the presence vs. absence of *so* (abbreviated *Uey* and *Uey; so^1^*, respectively) or a change in expression in leg discs co-overexpressing *eya* and *so* compared to wild type leg discs (abbreviated *Ueya+so*). Genes meeting these criteria were then intersected with the list of genes that have So ChIP-seq peaks to identify putative direct targets of So. Intersection of these data sets identifies a total of 810 genes that show differential expression between *Uey* and *Uey; so^1^* leg discs, between wild-type and *Ueya+so* leg discs, or both (Table S2).

So has been suggested to function as a transcriptional activator as well as transcriptional repressor during development [43], [44]. Consistent with this, the 810 genes include ones that respond positively as well as ones that respond negatively to So. 468 genes (57.8%) are putative positive targets of So – genes that are downregulated in absence of *so*, upregulated in presence of ectopic *so*, or both. In contrast, 290 genes (35.8%) are candidate negative So targets, which show higher expression in *so* mutant discs, lower expression in *so*-overexpressing discs, or both. We refer to the remaining 52 genes (6.4%) as “ambiguous” genes, because they appear positively regulated by So in the *Uey* vs. *Uey; so^1^* assay and negatively regulated in the wt vs. *Ueya+so* assay, or vice versa (Table S2). The regulation of the “ambiguous” genes by So may be context-specific or indirect. Three genes (*nonA, CG2225,* and *Gαq*) are both up- and downregulated in *Uey* vs. *Uey; so^1^*, possibly reflecting different regulation of distinct splice isoforms of each gene by So (for each of these three genes, distinct Affymetrix probes show up- and downregulation in response to loss of *so*).

Among the 810 candidate So targets, 460 show differential expression in response to loss of *so* (*Uey* vs. *Uey; so^1^*) and 444 respond to gain of ectopic *so* (wt vs. *Ueya+so*), with a 94-gene overlap between the two sets. Among the 486 genes that only respond positively to So, 257 are more highly expressed in *Uey* vs. *Uey; so^1^*, and 244 are upregulated in *Ueya+so* relative to wt, with 33 genes (7.1% of all positively regulated genes) that respond positively to So in both loss- and gain-of-function conditions. The 290 genes that act only as negative targets of So include 151 genes that show lower expression in *Uey* than in *Uey; so^1^*, and 149 genes with lower expression in *Ueya+so* than in wt, with 10 genes (3.4% all negative targets) that respond negatively to So in both microarray conditions (this summary excludes the “ambiguous” genes that respond positively to So in one assay and negatively in another) (Table S2).

### Putative targets of So during eye development

The 468 genes that are activated by So in the microarray datasets include many previously shown to play an important role during eye development. These eye genes include previously known direct targets of So such as *ey*, *ato*, and *lz,* as well as genes encoding a variety of transcription factors and co-factors, signaling pathway components, cytoskeletal components, and chromatin modifying proteins. The transcription factors and co-factors include retinal determination genes such as *Optix, eya, distal antenna* (*dan*), and *distal antenna related* (*danr*) [21], [39], [45], as well as several transcription factors needed for differentiation of retinal cells posterior to the MF such as *senseless* (*sens*) and *glass* (*gl*) [46], [47]. The putative So targets in signaling pathways include genes such as the receptor tyrosine kinase *sevenless* (*sev*, required in the R7 photoreceptor), *phyllopod* (EGFR pathway target and antagonist of Notch signaling), *roughoid*, and *pointed* (EGFR signaling) [48], [49], [50], [51], [52], [53], [54]. The putative So targets *Arpc1* (*Actin-related protein 2/3 complex subunit 1*) and *roughest* (*rest*) are involved in pupal retinal development [55], [56] while putative So targets involved in chromatin structure such as *Caf1* (*Chromatin assembly factor 1 subunit*) and *lola* (*longitudinals lacking*) have published eye phenotypes [57], [58].

## Discussion

The homeodomain transcription factor So plays vital roles in *Drosophila* eye development, yet only a few of its direct targets are currently known. Using ChIP-seq, we have mapped So-enriched regions throughout the genome in third instar larval eye discs. We found 7,566 So-enriched regions, which map to approximately 5,952 genes, including previously characterized So targets in the eye. The genes that map to the 10% most significant So peaks are enriched in GO categories that are relevant to eye development, such as sensory organ development, neuron differentiation, imaginal disc pattern formation, and transcription regulation. The ChIP-seq data set greatly expands our list of putative So targets in the eye, and it suggests that So regulates multiple processes during eye development.

### So peaks show enrichment for transcription factor binding motifs

Many transcription factors, including So, regulate their targets by binding cooperatively with other transcriptional regulators [6], [10]. To identify putative So-interacting transcriptional regulators in the eye, we performed *de novo* motif analysis of the entire So peak set using the online software RSAT [15]. As predicted, we observed enrichment for AGATAC, which closely matches the consensus So motif [11]. We also found enrichment for other transcription factor consensus motifs, some of which are known to be active in the eye disc. One of the enriched motifs corresponds to Optix, a So paralog and an RD network member that is expressed anterior to the MF in the eye disc [21]. The So peak set also shows enrichment for the consensus motif of Iroquois complex (Iro-C) homeodomain transcription factors – Mirror (Mirr), Araucan (Ara), and Caupolican (Caup). These factors, expressed in the dorsal half of the early eye disc, establish dorsal/ventral (DV) polarity in the eye disc, and activate Notch signaling at the DV midline that is necessary for eye disc growth [59], [60]. Whether So and Iro-C interact functionally to regulate target genes is unclear, but this could potentially happen in the dorsal-anterior quadrant of the third instar eye disc, where So and Iro-C are coexpressed.

Our motif analysis also suggests a putative link between So and transcriptional response to the hormone ecdysone, which triggers larval molting and pupariation in *Drosophila,* and regulates MF progression and the cell cycle in the eye disc [61], [62]. The So ChIP-seq peak set is enriched for the consensus motif for Br-Z1, an isoform of the Broad transcription factor, which is expressed posterior to the MF and is transcriptionally activated in response to ecdysone [63], as well as for Ultraspiracle (Usp), which forms an ecdysone-responsive heterodimer with the Ecdysone Receptor (EcR) [25]. Usp and Br appear to have opposite effects on eye development: while Br promotes MF progression, Usp antagonizes it, and Usp represses *br* expression in the eye disc [64], [65]. The enrichment of Br-Z1 and Usp motifs in the So peak set, as well as the presence of the term *Response to ecdysone* in the GO analysis of genes with highly enriched So peaks, suggests a possible role for So in regulating the response to ecdysone, a possibility that will need to be tested in future studies.

Another highly enriched motif among So peaks is the DNA Replication Element (DRE), which binds the DRE factor (Dref), an activator of DNA replication and cell proliferation [66]. In the eye disc Dref is expressed predominantly in proliferating cells anterior to the MF, and DREs are enriched in the promoter regions of genes that show high expression anterior to the MF [67]. Dref can also bind a chromatin boundary element [68], and several chromatin remodeling factors interact genetically with Dref in the eye [69]. These data suggest that Dref may play a role in chromatin remodeling. Trithorax-like (Trl, a.k.a. GAGA Factor) is also a chromatin remodeling protein active in the eye that shows consensus motif enrichment in the So peak set [70]. Altogether, these data suggest that So may contribute to regulating the cell cycle and/or chromatin state in the eye disc by acting together with Dref and Trl. This is consistent with the loss-of-function phenotypes of *so* mutant tissue, which shows defects in cell proliferation [5]. Future studies will be needed to test the functional significance of motif enrichment in the So peak set.

### So binds to genes predicted to function in eye development

The top 10% most significant So peaks map to genes that are enriched in GO categories pertaining to eye and neuronal development, such as *Imaginal disc development*, *Sensory organ development/Compound eye development*, *Neuron differentiation*, and *Regulation of photoreceptor cell differentiation*. Most of the previously known So target genes encode transcription factors that drive successive stages of eye development: *ey,* which is necessary for eye specification and for initial expression of So, which then regulates *ey* in a feedback loop [71]; *dac,* which is necessary for the onset of differentiation in the eye disc; *ato*, which is required for the specification of the R8, the first photoreceptor that forms in the eye; and *lz* and its target *pros*, both of which regulate the differentiation of specific cell types. Consistent with these previous data, highly So-enriched genes are over-represented in the GO term *Regulation of transcription*, as well as InterPro protein domains that occur in transcription factors, such as homeobox and zinc finger (Table S1).

### Putative direct targets of So

Of the many genes enriched for So binding in eye discs, only a fraction are expected to be true So targets. In order to identify candidate direct targets of So in eye development, we have overlapped the set of genes enriched for So binding with genes that are differentially regulated in response to loss or gain of *so*, using genome-wide expression data sets. Ectopic overexpression of genes that encode transcriptional regulators in the RD network, such as *so* and *ey*, is sufficient to trigger ectopic eye formation in *Drosophila* [40], [41]. A previous study analyzed microarray gene expression changes in response to ectopic eye induction in the leg imaginal disc ([32] and our unpublished data). Based on these previous data, we assembled two lists of candidate So target genes. The first list contains genes that are regulated by ectopic *ey* (*UAS-ey,* abbreviated *Uey*) in a *so*-dependent manner (i.e., genes that show differential expression between *Uey* leg discs and *Uey; so^1^* leg discs). The second list includes genes that respond to ectopic overexpression of *so* and its binding partner *eya* in the leg disc (differential expression between wild-type and *Ueya+so* leg discs).

We identified 810 genes that have a So ChIP-seq peak and are differentially regulated in one or both of the above conditions (*Uey* vs. *Uey; so^1^* and wild-type vs. *Ueya+so*). 460 genes respond to loss of *so* (differential expression between *Uey* and *Uey; so^1^*), and 444 genes respond to gain of *so* (difference in expression between wt and *Ueya+so* leg discs). The overlap between the *Uey; so^1^* and the *Ueya+so* gene lists is 94 genes (11.6%). Although both *Uey* and *Ueya+so* are capable of inducing ectopic eye formation, ectopic *ey* is a much more potent inducer of retinal fate than ectopic *eya*+*so*, and this may account, at least in part, for the small overlap between these two datasets [40], [72]. Hence, *Uey* and *Ueya+so* appear to regulate largely distinct sets of genes in the leg disc. Our approach likely resulted in many false negatives, as ectopic overexpression of RD genes does not fully recapitulate normal eye development. Notably, the microarray used RNA prepared from the whole leg disc, but *Uey* triggers ectopic retinal development in only a small fraction of the leg disc cells; hence, a gene that is only moderately upregulated in the minority of cells that take on an ectopic eye fate is unlikely to be detected as significantly upregulated in RNA prepared from whole *Uey* leg discs. Nonetheless, loss of *so* in *Uey* leg discs leads to reduced expression of the previously known So targets *lz* and *ato*, indicating that many gene expression changes observed in ectopic eye induction may be relevant to normal eye development. GO analysis of the genes that appear to be activated by So based on microarray data shows enrichment for terms associated with eye, imaginal disc, and neuron development. This is consistent with the ability of *Ueya+so* to induce ectopic eyes, and the requirement for *so* in *Uey-*mediated ectopic eye induction. The *Ueya+so* responsive genes in the leg disc include genes previously known to be required in eye development, such as *danr,* which is expressed anterior to and within the MF and is required for the onset of photoreceptor differentiation. Danr is part of the RD network; it interacts physically with the RD proteins Ey and Dac, and its overexpression can trigger ectopic eye formation in the antenna [73].

### Novel targets of So in eye development

In an attempt to uncover novel targets of So that are necessary for eye development, we obtained fly stocks with mutations in novel, So-enriched genes, and tested the requirement for these genes in eye development using the *FLP/FRT* system. We identified eight genes that appear to be required for eye development, as their loss in the eye disc leads to reduced, misshapen, and/or disorganized eyes. A loss-of-function mutation in one additional gene, *kkv*, causes a pharate lethal phenotype with severe reduction in head size, suggesting that it may act early in development, upstream of *so*.

When choosing putative So target genes, we made the assumption that the gene nearest to or overlapping a So peak is the putative target, an assumption that may not always be correct. A transcription factor may regulate genes many kilobases away, through long-distance interactions mediated by chromatin looping [74]. If a small gene is nested in an intron of a larger gene that has a So peak, the small gene rather than the large gene may be the true So target. Moreover, if a So peak maps to an intergenic region, we automatically map it to the nearest gene in either direction, which is not necessarily the true target.

In addition to potential inaccuracies in mapping So peaks to target genes, there are several other reasons why only nine genes tested resulted in eye or head phenotypes. First, we limited ourselves to already existing alleles that were available from stock collections. Many genes that would have been interesting to test, such as novel So-enriched genes that show differential regulation during ectopic eye induction (Table S2), do not have publicly available mutant alleles. Second, most of the alleles we ordered were transposon insertions in an intron or UTR of the gene of interest that were likely to disrupt gene function only weakly or not at all. Availability of stronger alleles may have resulted in eye phenotypes with a larger percentage of the genes tested. Third, as discussed above, So does not necessarily regulate the gene to which it binds, as the So peak may be an experimental artifact, reflect nonfunctional binding, or be regulating an adjacent gene. For example, the novel gene *CG7576* overlaps a large So peak, yet it has no phenotype in the eye, possibly because the So peak reflects regulation of the nearby gene *glass*, known to be necessary for eye differentiation [47].

Despite the caveats above, we identified eight novel genes that appear to regulate eye development downstream of So. The eight mutants show a range of phenotypes, from a disorganized but full-sized eye to almost complete loss of eye, reflecting the requirement for So at multiple stages of eye development, from cell survival and MF initiation to differentiation [3]. One putative direct So target gene is *oocyte maintenance defects* (*omd*), predicted to encode an RNA processing protein. Little is currently known about the function of *omd*, although previous studies suggest *omd* interacts with the Dpp pathway [75] and regulates larval neurogenesis [37], suggesting that it may function downstream of So in the developing photoreceptors. Detailed characterization of *omd* and other novel genes, as well as their regulatory interaction with So, awaits further studies. In summary, our work identifies a wealth of putative direct So targets that will expand our understanding of So-mediated transcriptional regulation in development.

ChIP-seq data suggest the presence of multiple feed-forward loops. For example, previous studies have shown that So and Ey positively regulate each other in early eye development [8], [76], and subsequently So and Ey together initiate *ato* expression [10]. Ato is necessary for the specification of the R8 photoreceptor, where it initiates the expression of *senseless* (*sens*), which encodes a zinc finger transcription factor necessary for R8 development [46], [77]. *sens* has a So peak, and *sens* induction by ectopic *ey* in the leg disc requires *so* (unpublished data), suggesting that So may directly regulate *sens.* Such a result would be a three-level feed-forward loop, whereby Ey and So activate each other, So and Ey activate *ato*, and So and Ato activate *sens.* Similarly, in differentiating cells posterior to the MF, So activates *lz* [9], which encodes a transcription factor that regulates multiple downstream targets, including the direct So target *pros* [6]. Lz also activates the transcription factors *D-Pax2* in cone cell precursors and *Bar-H1/2* in R1/6 photoreceptor precursors [78], [79]. *D-Pax2, Bar-H1,* and *Bar-H2* all have So ChIP-seq peaks, suggesting the possibility of another feed-forward loop: So activates *lz* and then cooperates with Lz in activating downstream transcriptional regulators in different cell types posterior to the MF.

Another intriguing possibility is that So may regulate the expression of cytoskeleton and cell junction components, many of which are encoded by genes that have So peaks, and some of which appear to be regulated by So in ectopic eye formation. While many cell junction components are expressed uniformly throughout the developing eye disc, there are examples of dynamic regulation of cell junction protein expression in the eye: immediately posterior to the MF, the transcription factor Ato and the Egfr signaling pathway together promote high expression of the cell junction protein-encoding genes *shotgun* and *armadillo* in nascent ommatidial clusters [80]. *Drosophila* So has not been shown to control the expression of cytoskeleton component genes, but the murine So homolog Six1 directly activates *Vil2,* which encodes the actin cytoskeleton regulator Ezrin, thus promoting cell motility and metastasis in cancer [81]. Since So-expressing cells of the eye disc undergo dynamic shape changes associated with the passage of the MF and the formation of ommatidial clusters, and the optic lobe does not invaginate correctly in *so* mutant embryos [3], [4], it is possible that one of the functions of So in development is to regulate the expression of cell adhesion and cytoskeleton molecules that control morphogenesis. Our results provide the basis for many specific hypotheses concerning eye development.

## Supporting Information

Table S1 **Gene Ontology (GO) terms associated with genes highly enriched for So binding in the eye disc.**
(DOCX)

Table S2 **Putative direct targets of So based on transcriptional response to loss or gain of** ***so*** **in ectopic eye.** All genes listed have a So ChIP-seq peak, and show significant change in expression in response to either loss of *so* in ectopic eye (*Uey* vs. *Uey;so^1^*) or ectopic overexpression of *so* and its cofactor *eya* (*Ueya+so* vs. wt). Depending on their response to So, genes are classified as POS (positively regulated, i.e. activated, by So), NEG (negatively regulated, i.e. repressed, by So), or AMB (ambiguous – respond positively to So in one assay and negatively in another).
(XLSX)

Dataset S1 **So ChIP-seq peaks in the eye disc.** Each peak has a unique index number, which reflects its P-value (lower index number corresponds to higher P-value and hence more significant peak). P-value  = 50 corresponds to P = 10^−5^. The coordinates of each peak are given, as is the identity of the nearest gene and the nearest transcription start site (TSS). The GENE_STATUS column indicates whether a peak is intragenic (GENE_OVERLAP) or intergenic (GENE_CLOSE). For intergenic peaks, the distance to nearest gene is given in the GENE_DIS column. The distance to the nearest TSS is in the TSS_DIS column. Note that the nearest TSS may belong to a gene different from the one listed in the GENE column.
(XLSX)

## References

[1] Pappu KS, Mardon G (2004) Genetic control of retinal specification and determination in Drosophila. Int J Dev Biol 48: 913–924. 15558482 10.1387/ijdb.041875kp

[2] Ready DF, Hanson TE, Benzer S (1976) Development of the Drosophila retina, a neurocrystalline lattice. Dev Biol 53: 217–240. 825400 10.1016/0012-1606(76)90225-6

[3] Cheyette BN, Green PJ, Martin K, Garren H, Hartenstein V, et al (1994) The Drosophila sine oculis locus encodes a homeodomain-containing protein required for the development of the entire visual system. Neuron 12: 977–996. 7910468 10.1016/0896-6273(94)90308-5

[4] Serikaku MA, O′Tousa JE (1994) sine oculis is a homeobox gene required for Drosophila visual system development. Genetics 138: 1137–1150. 7896096 10.1093/genetics/138.4.1137 PMC1206253

[5] Pignoni F, Hu B, Zavitz KH, Xiao J, Garrity PA, et al (1997) The eye-specification proteins So and Eya form a complex and regulate multiple steps in Drosophila eye development. Cell 91: 881–891. 9428512 10.1016/s0092-8674(00)80480-8

[6] Hayashi T, Xu C, Carthew RW (2008) Cell-type-specific transcription of prospero is controlled by combinatorial signaling in the Drosophila eye. Development 135: 2787–2796. 18635611 10.1242/dev.006189 PMC2923442

[7] Pappu KS, Chen R, Middlebrooks BW, Woo C, Heberlein U, et al (2003) Mechanism of hedgehog signaling during Drosophila eye development. Development 130: 3053–3062. 12756186 10.1242/dev.00534

[8] Pauli T, Seimiya M, Blanco J, Gehring WJ (2005) Identification of functional sine oculis motifs in the autoregulatory element of its own gene, in the eyeless enhancer and in the signalling gene hedgehog. Development 132: 2771–2782. 15901665 10.1242/dev.01841

[9] Yan H, Canon J, Banerjee U (2003) A transcriptional chain linking eye specification to terminal determination of cone cells in the Drosophila eye. Dev Biol 263: 323–329. 14597205 10.1016/j.ydbio.2003.08.003

[10] Zhang T, Ranade S, Cai CQ, Clouser C, Pignoni F (2006) Direct control of neurogenesis by selector factors in the fly eye: regulation of atonal by Ey and So. Development 133: 4881–4889. 17108002 10.1242/dev.02669

[11] Jemc J, Rebay I (2007) Identification of transcriptional targets of the dual-function transcription factor/phosphatase eyes absent. Dev Biol 310: 416–429. 17714699 10.1016/j.ydbio.2007.07.024 PMC2075104

[12] Mutsuddi M, Chaffee B, Cassidy J, Silver SJ, Tootle TL, et al (2005) Using Drosophila to decipher how mutations associated with human branchio-oto-renal syndrome and optical defects compromise the protein tyrosine phosphatase and transcriptional functions of eyes absent. Genetics 170: 687–695. 15802522 10.1534/genetics.104.039156 PMC1450419

[13] Zhang Y, Liu T, Meyer CA, Eeckhoute J, Johnson DS, et al (2008) Model-based analysis of ChIP-Seq (MACS). Genome Biol 9: R137. 18798982 10.1186/gb-2008-9-9-r137 PMC2592715

[14] Dennis G Jr, Sherman BT, Hosack DA, Yang J, Gao W, et al (2003) DAVID: Database for Annotation, Visualization, and Integrated Discovery. Genome Biol 4: P3. 12734009 PMC193660

[15] Thomas-Chollier M, Herrmann C, Defrance M, Sand O, Thieffry D, et al (2012) RSAT peak-motifs: motif analysis in full-size ChIP-seq datasets. Nucleic Acids Res 40: e31. 22156162 10.1093/nar/gkr1104 PMC3287167

[16] Buszczak M, Paterno S, Lighthouse D, Bachman J, Planck J, et al (2007) The carnegie protein trap library: a versatile tool for Drosophila developmental studies. Genetics 175: 1505–1531. 17194782 10.1534/genetics.106.065961 PMC1840051

[17] Newsome TP, Asling B, Dickson BJ (2000) Analysis of Drosophila photoreceptor axon guidance in eye-specific mosaics. Development 127: 851–860. 10648243 10.1242/dev.127.4.851

[18] Tomlinson A, Ready DF (1987) Cell fate in the Drosophila ommatidium. Dev Biol 123: 264–275. 17985474 10.1016/0012-1606(87)90448-9

[19] Rogers EM, Brennan CA, Mortimer NT, Cook S, Morris AR, et al (2005) Pointed regulates an eye-specific transcriptional enhancer in the Drosophila hedgehog gene, which is required for the movement of the morphogenetic furrow. Development 132: 4833–4843. 16207753 10.1242/dev.02061

[20] Xu C, Kauffmann RC, Zhang J, Kladny S, Carthew RW (2000) Overlapping activators and repressors delimit transcriptional response to receptor tyrosine kinase signals in the Drosophila eye. Cell 103: 87–97. 11051550 10.1016/s0092-8674(00)00107-0

[21] Seimiya M, Gehring WJ (2000) The Drosophila homeobox gene optix is capable of inducing ectopic eyes by an eyeless-independent mechanism. Development 127: 1879–1886. 10751176 10.1242/dev.127.9.1879

[22] McNeill H, Yang CH, Brodsky M, Ungos J, Simon MA (1997) mirror encodes a novel PBX-class homeoprotein that functions in the definition of the dorsal-ventral border in the Drosophila eye. Genes Dev 11: 1073–1082. 9136934 10.1101/gad.11.8.1073

[23] Dominguez M, de Celis JF (1998) A dorsal/ventral boundary established by Notch controls growth and polarity in the Drosophila eye. Nature 396: 276–278. 9834035 10.1038/24402

[24] Hirose F, Yamaguchi M, Kuroda K, Omori A, Hachiya T, et al (1996) Isolation and characterization of cDNA for DREF, a promoter-activating factor for Drosophila DNA replication-related genes. J Biol Chem 271: 3930–3937. 8632015 10.1074/jbc.271.7.3930

[25] Yao TP, Forman BM, Jiang Z, Cherbas L, Chen JD, et al (1993) Functional ecdysone receptor is the product of EcR and Ultraspiracle genes. Nature 366: 476–479. 8247157 10.1038/366476a0

[26] Huang DW SB, Lempicki RA (2009) Systematic and integrative analysis of large gene lists using DAVID Bioinformatics Resources. Nat Protocols 4: 44–57. 19131956 10.1038/nprot.2008.211

[27] Huang DW SB, Lempicki RA (2009) Bioinformatics enrichment tools: paths toward the comprehensive functional analysis of large gene lists. Nucleic Acids Res 37: 1–13. 19033363 10.1093/nar/gkn923 PMC2615629

[28] Mardon G, Solomon NM, Rubin GM (1994) dachshund encodes a nuclear protein required for normal eye and leg development in Drosophila. Development 120: 3473–3486. 7821215 10.1242/dev.120.12.3473

[29] Pappu KS, Ostrin EJ, Middlebrooks BW, Sili BT, Chen R, et al (2005) Dual regulation and redundant function of two eye-specific enhancers of the Drosophila retinal determination gene dachshund. Development 132: 2895–2905. 15930118 10.1242/dev.01869

[30] Liu YH, Jakobsen JS, Valentin G, Amarantos I, Gilmour DT, et al (2009) A systematic analysis of Tinman function reveals Eya and JAK-STAT signaling as essential regulators of muscle development. Dev Cell 16: 280–291. 19217429 10.1016/j.devcel.2009.01.006

[31] Doroquez DB, Rebay I (2006) Signal integration during development: mechanisms of EGFR and Notch pathway function and cross-talk. Crit Rev Biochem Mol Biol 41: 339–385. 17092823 10.1080/10409230600914344

[32] Ostrin EJ, Li Y, Hoffman K, Liu J, Wang K, et al (2006) Genome-wide identification of direct targets of the Drosophila retinal determination protein Eyeless. Genome Res 16: 466–476. 16533912 10.1101/gr.4673006 PMC1457028

[33] Cruz C, Glavic A, Casado M, de Celis JF (2009) A gain-of-function screen identifying genes required for growth and pattern formation of the Drosophila melanogaster wing. Genetics 183: 1005–1026. 19737745 10.1534/genetics.109.107748 PMC2778956

[34] Heberlein U, Wolff T, Rubin GM (1993) The TGF beta homolog dpp and the segment polarity gene hedgehog are required for propagation of a morphogenetic wave in the Drosophila retina. Cell 75: 913–926. 8252627 10.1016/0092-8674(93)90535-x

[35] Ezzeddine N, Chen J, Waltenspiel B, Burch B, Albrecht T, et al (2011) A subset of Drosophila integrator proteins is essential for efficient U7 snRNA and spliceosomal snRNA 3′-end formation. Mol Cell Biol 31: 328–341. 21078872 10.1128/MCB.00943-10 PMC3019983

[36] Rutkowski RJ, Warren WD (2009) Phenotypic analysis of deflated/Ints7 function in Drosophila development. Dev Dyn 238: 1131–1139. 19326441 10.1002/dvdy.21922

[37] Neumuller RA, Richter C, Fischer A, Novatchkova M, Neumuller KG, et al (2011) Genome-wide analysis of self-renewal in Drosophila neural stem cells by transgenic RNAi. Cell Stem Cell 8: 580–593. 21549331 10.1016/j.stem.2011.02.022 PMC3093620

[38] Sousa-Nunes R, Chia W, Somers WG (2009) Protein phosphatase 4 mediates localization of the Miranda complex during Drosophila neuroblast asymmetric divisions. Genes Dev 23: 359–372. 19204120 10.1101/gad.1723609 PMC2648543

[39] Bonini NM, Bui QT, Gray-Board GL, Warrick JM (1997) The Drosophila eyes absent gene directs ectopic eye formation in a pathway conserved between flies and vertebrates. Development 124: 4819–4826. 9428418 10.1242/dev.124.23.4819

[40] Halder G, Callaerts P, Gehring WJ (1995) Induction of ectopic eyes by targeted expression of the eyeless gene in Drosophila. Science 267: 1788–1792. 7892602 10.1126/science.7892602

[41] Weasner B, Salzer C, Kumar JP (2007) Sine oculis, a member of the SIX family of transcription factors, directs eye formation. Dev Biol 303: 756–771. 17137572 10.1016/j.ydbio.2006.10.040 PMC2719711

[42] Halder G, Callaerts P, Flister S, Walldorf U, Kloter U, et al (1998) Eyeless initiates the expression of both sine oculis and eyes absent during Drosophila compound eye development. Development 125: 2181–2191. 9584118 10.1242/dev.125.12.2181

[43] Silver SJ, Davies EL, Doyon L, Rebay I (2003) Functional dissection of eyes absent reveals new modes of regulation within the retinal determination gene network. Mol Cell Biol 23: 5989–5999. 12917324 10.1128/MCB.23.17.5989-5999.2003 PMC180989

[44] Anderson AM, Weasner BM, Weasner BP, Kumar JP (2012) Dual transcriptional activities of SIX proteins define their roles in normal and ectopic eye development. Development 139: 991–1000. 22318629 10.1242/dev.077255 PMC3274360

[45] Curtiss J, Heilig JS (1997) Arrowhead encodes a LIM homeodomain protein that distinguishes subsets of Drosophila imaginal cells. Dev Biol 190: 129–141. 9331336 10.1006/dbio.1997.8659

[46] Frankfort BJ, Nolo R, Zhang Z, Bellen H, Mardon G (2001) senseless repression of rough is required for R8 photoreceptor differentiation in the developing Drosophila eye. Neuron 32: 403–414. 11709152 10.1016/s0896-6273(01)00480-9 PMC3122332

[47] Moses K, Ellis MC, Rubin GM (1989) The glass gene encodes a zinc-finger protein required by Drosophila photoreceptor cells. Nature 340: 531–536. 2770860 10.1038/340531a0

[48] Brunner D, Ducker K, Oellers N, Hafen E, Scholz H, et al (1994) The ETS domain protein pointed-P2 is a target of MAP kinase in the sevenless signal transduction pathway. Nature 370: 386–389. 8047146 10.1038/370386a0

[49] Chang HC, Solomon NM, Wassarman DA, Karim FD, Therrien M, et al (1995) phyllopod functions in the fate determination of a subset of photoreceptors in Drosophila. Cell 80: 463–472. 7888014 10.1016/0092-8674(95)90497-2

[50] Nagaraj R, Banerjee U (2009) Regulation of Notch and Wingless signalling by phyllopod, a transcriptional target of the EGFR pathway. EMBO J 28: 337–346. 19153610 10.1038/emboj.2008.286 PMC2646148

[51] O′Neill EM, Rebay I, Tjian R, Rubin GM (1994) The activities of two Ets-related transcription factors required for Drosophila eye development are modulated by the Ras/MAPK pathway. Cell 78: 137–147. 8033205 10.1016/0092-8674(94)90580-0

[52] Simon MA, Bowtell DD, Rubin GM (1989) Structure and activity of the sevenless protein: a protein tyrosine kinase receptor required for photoreceptor development in Drosophila. Proc Natl Acad Sci U S A 86: 8333–8337. 2682647 10.1073/pnas.86.21.8333 PMC298275

[53] Tomlinson A, Ready DF (1987) Neuronal differentiation in Drosophila ommatidium. Dev Biol 120: 366–376. 17985475 10.1016/0012-1606(87)90239-9

[54] Wasserman JD, Urban S, Freeman M (2000) A family of rhomboid-like genes: Drosophila rhomboid-1 and roughoid/rhomboid-3 cooperate to activate EGF receptor signaling. Genes Dev 14: 1651–1663. 10887159 PMC316740

[55] Zallen JA, Cohen Y, Hudson AM, Cooley L, Wieschaus E, et al (2002) SCAR is a primary regulator of Arp2/3-dependent morphological events in Drosophila. J Cell Biol 156: 689–701. 11854309 10.1083/jcb.200109057 PMC2174092

[56] Araujo H, Machado LC, Octacilio-Silva S, Mizutani CM, Silva MJ, et al (2003) Requirement of the roughest gene for differentiation and time of death of interommatidial cells during pupal stages of Drosophila compound eye development. Mech Dev 120: 537–547. 12782271 10.1016/s0925-4773(03)00040-6

[57] Anderson AE, Karandikar UC, Pepple KL, Chen Z, Bergmann A, et al (2011) The enhancer of trithorax and polycomb gene Caf1/p55 is essential for cell survival and patterning in Drosophila development. Development 138: 1957–1966. 21490066 10.1242/dev.058461 PMC3082301

[58] Zheng L, Carthew RW (2008) Lola regulates cell fate by antagonizing Notch induction in the Drosophila eye. Mech Dev 125: 18–29. 18053694 10.1016/j.mod.2007.10.007 PMC2782576

[59] Cavodeassi F, Diez Del Corral R, Campuzano S, Dominguez M (1999) Compartments and organising boundaries in the Drosophila eye: the role of the homeodomain Iroquois proteins. Development 126: 4933–4942. 10529412 10.1242/dev.126.22.4933

[60] Dominguez M, Wasserman JD, Freeman M (1998) Multiple functions of the EGF receptor in Drosophila eye development. Curr Biol 8: 1039–1048. 9768358 10.1016/s0960-9822(98)70441-5

[61] Brennan CA, Ashburner M, Moses K (1998) Ecdysone pathway is required for furrow progression in the developing Drosophila eye. Development 125: 2653–2664. 9636080 10.1242/dev.125.14.2653

[62] Riddiford LM (1993) Hormone receptors and the regulation of insect metamorphosis. Receptor 3: 203–209. 8167571

[63] Brennan CA, Li TR, Bender M, Hsiung F, Moses K (2001) Broad-complex, but not ecdysone receptor, is required for progression of the morphogenetic furrow in the Drosophila eye. Development 128: 1–11. 11092806 10.1242/dev.128.1.1

[64] Ghbeish N, McKeown M (2002) Analyzing the repressive function of ultraspiracle, the Drosophila RXR, in Drosophila eye development. Mech Dev 111: 89–98. 11804781 10.1016/s0925-4773(01)00610-4

[65] Zelhof AC, Ghbeish N, Tsai C, Evans RM, McKeown M (1997) A role for ultraspiracle, the Drosophila RXR, in morphogenetic furrow movement and photoreceptor cluster formation. Development 124: 2499–2506. 9216992 10.1242/dev.124.13.2499

[66] Ohno K, Hirose F, Sakaguchi K, Nishida Y, Matsukage A (1996) Transcriptional regulation of the Drosophila CycA gene by the DNA replication-related element (DRE) and DRE binding factor (DREF). Nucleic Acids Res 24: 3942–3946. 8918795 10.1093/nar/24.20.3942 PMC146190

[67] Jasper H, Benes V, Atzberger A, Sauer S, Ansorge W, et al (2002) A genomic switch at the transition from cell proliferation to terminal differentiation in the Drosophila eye. Dev Cell 3: 511–521. 12408803 10.1016/s1534-5807(02)00297-6

[68] Hart CM, Cuvier O, Laemmli UK (1999) Evidence for an antagonistic relationship between the boundary element-associated factor BEAF and the transcription factor DREF. Chromosoma 108: 375–383. 10591997 10.1007/s004120050389

[69] Hirose F, Ohshima N, Shiraki M, Inoue YH, Taguchi O, et al (2001) Ectopic expression of DREF induces DNA synthesis, apoptosis, and unusual morphogenesis in the Drosophila eye imaginal disc: possible interaction with Polycomb and trithorax group proteins. Mol Cell Biol 21: 7231–7242. 11585906 10.1128/MCB.21.21.7231-7242.2001 PMC99898

[70] Farkas G, Gausz J, Galloni M, Reuter G, Gyurkovics H, et al (1994) The Trithorax-like gene encodes the Drosophila GAGA factor. Nature 371: 806–808. 7935842 10.1038/371806a0

[71] Atkins M, Jiang Y, Sansores-Garcia L, Jusiak B, Halder G, et al (2013) Dynamic rewiring of the Drosophila retinal determination network switches its function from selector to differentiation. PLoS Genet 9: e1003731. 24009524 10.1371/journal.pgen.1003731 PMC3757064

[72] Pignoni F, Zipursky SL (1997) Induction of Drosophila eye development by decapentaplegic. Development 124: 271–278. 9053304 10.1242/dev.124.2.271

[73] Curtiss J, Burnett M, Mlodzik M (2007) distal antenna and distal antenna-related function in the retinal determination network during eye development in Drosophila. Dev Biol 306: 685–702. 17493605 10.1016/j.ydbio.2007.04.006 PMC1986786

[74] Farnham PJ (2009) Insights from genomic profiling of transcription factors. Nat Rev Genet 10: 605–616. 19668247 10.1038/nrg2636 PMC2846386

[75] Cai C (2006) Oocyte maintenance defects restricts Dpp responsive cells to the stem cell niche in the Drosophila germarium. A Dros Res Conf 47: 466C.

[76] Niimi T, Seimiya M, Kloter U, Flister S, Gehring WJ (1999) Direct regulatory interaction of the eyeless protein with an eye-specific enhancer in the sine oculis gene during eye induction in Drosophila. Development 126: 2253–2260. 10207149 10.1242/dev.126.10.2253

[77] Jarman AP, Grell EH, Ackerman L, Jan LY, Jan YN (1994) Atonal is the proneural gene for Drosophila photoreceptors. Nature 369: 398–400. 8196767 10.1038/369398a0

[78] Flores GV, Duan H, Yan H, Nagaraj R, Fu W, et al (2000) Combinatorial signaling in the specification of unique cell fates. Cell 103: 75–85. 11051549 10.1016/s0092-8674(00)00106-9

[79] Lim J, Choi KW (2004) Drosophila eye disc margin is a center for organizing long-range planar polarity. Genesis 39: 26–37. 15124224 10.1002/gene.20022

[80] Brown KE, Baonza A, Freeman M (2006) Epithelial cell adhesion in the developing Drosophila retina is regulated by Atonal and the EGF receptor pathway. Dev Biol 300: 710–721. 16963016 10.1016/j.ydbio.2006.08.003

[81] Yu Y, Davicioni E, Triche TJ, Merlino G (2006) The homeoprotein six1 transcriptionally activates multiple protumorigenic genes but requires ezrin to promote metastasis. Cancer Res 66: 1982–1989. 16488997 10.1158/0008-5472.CAN-05-2360

[82] Johnson K, Knust E, Skaer H (1999) bloated tubules (blot) encodes a Drosophila member of the neurotransmitter transporter family required for organisation of the apical cytocortex. Dev Biol 212: 440–454. 10433833 10.1006/dbio.1999.9351

[83] Schuldiner O, Berdnik D, Levy JM, Wu JS, Luginbuhl D, et al (2008) piggyBac-based mosaic screen identifies a postmitotic function for cohesin in regulating developmental axon pruning. Dev Cell 14: 227–238. 18267091 10.1016/j.devcel.2007.11.001 PMC2268086

[84] Spradling AC, Stern D, Beaton A, Rhem EJ, Laverty T, et al (1999) The Berkeley Drosophila Genome Project gene disruption project: Single P-element insertions mutating 25% of vital Drosophila genes. Genetics 153: 135–177. 10471706 10.1093/genetics/153.1.135 PMC1460730

[85] Bellen HJ, Levis RW, Liao G, He Y, Carlson JW, et al (2004) The BDGP gene disruption project: single transposon insertions associated with 40% of Drosophila genes. Genetics 167: 761–781. 15238527 10.1534/genetics.104.026427 PMC1470905

