# Supplementary material for: Regulation of Drosophila Eye Development by the Transcription Factor Sine oculis
Source: PLoS One. 2014 Feb 25;9(2):e89695. doi: 10.1371/journal.pone.0089695 (PMC3934907; doi:10.1371/journal.pone.0089695)
Supplement: Table S1 — Gene Ontology (GO) terms associated with genes highly enriched for So binding in the eye disc. (DOCX) [file pone.0089695.s001.docx]

**Table S1. GO terms for biological processes enriched in genes associated with the top 10% of So ChIP-seq peaks.** Enrichment Score >1.3 corresponds to P<0.05.

| **GO Term – Biological Process** | **Enrichment Score** |
| --- | --- |
| Imaginal disc development | 29.32 |
| Neuron differentiation | 14.84 |
| Regulation of transcription | 14.1 |
| Epithelium development/Tissue morphogenesis | 11.69 |
| Sensory organ development/Compound eye development | 9.94 |
| Imaginal disc pattern formation | 8.83 |
| Gland development | 8.13 |
| Establishment or maintenance of cell polarity | 7.77 |
| Formation of anatomical boundary | 7.18 |
| Enzyme linked receptor protein signaling pathway | 6.22 |
| Ovarian follicle cell development | 5.92 |
| Cell migration | 5.79 |
| Respiratory system development | 5.66 |
| Negative regulation of cell differentiation | 5.61 |
| Regulation of transcription from RNA Pol II promoter | 5.48 |
| Negative regulation of cell fate specification | 5.12 |
| Negative regulation of transcription from RNA Pol II promoter | 5.01 |
| Cell adhesion | 4.95 |
| Leg disc development | 4.63 |
| Proximal/distal pattern formation | 4.03 |
| Anterior/posterior pattern formation, imaginal disc | 3.71 |
| Muscle attachment | 3.32 |
| Regulation of cell development | 3.26 |
| R7 cell differentiation | 3.16 |
| Regulation of growth | 2.82 |
| Regulation of cell cycle | 2.7 |
| Gut development | 2.69 |
| Ectoderm development | 2.6 |
| Neuromuscular process | 2.45 |
| Morphogenesis of follicular epithelium | 2.44 |
| Olfactory behavior | 2.42 |
| Multicellular organismal homeostasis | 2.37 |
| Epithelial cell differentiation | 2.31 |
| Cell-cell junction organization | 2.29 |
| Regulation of programmed cell death | 2.29 |
| Neuron recognition | 2.27 |
| Anterior/posterior pattern formation | 2.18 |
| Muscle cell differentiation | 2.13 |
| Second mitotic wave during compound eye morphogenesis | 2.03 |
| Female germ-line cyst encapsulation | 2.02 |
| Cellular component assembly involved in morphogenesis | 2.01 |
| Response to wounding | 1.98 |
| Striated muscle cell differentiation | 1.98 |
| Asymmetric protein localization | 1.84 |
| Regulation of photoreceptor cell differentiation | 1.83 |
| Torso signaling pathway | 1.78 |
| Developmental growth | 1.66 |
| Actomyosin structure organization | 1.63 |
| Protein kinase cascade | 1.57 |
| Response to organic substance | 1.47 |
| Regulation of neuron differentiation | 1.45 |
| Gland morphogenesis/Response to ecdysone | 1.43 |
| Smoothened signaling pathway | 1.35 |
| Pigmentation | 1.33 |
| **InterPro Protein Domains** | |
| Homeobox | 3.44 |
| Zinc finger, C2H2-type | 2.58 |
| Src homology-3 (SH3) domain | 2.56 |
| Basic helix-loop-helix (bHLH) dimerization region | 2.54 |
| Frizzled protein | 2.31 |
| Myb/SANT-like domain in Adf-1 (MADF) domain | 2.28 |
| Epidermal Growth Factor (EGF) | 2.27 |
| Fibronectin, type III | 2.26 |
| Sterile alpha motif-type | 1.87 |
| Six-bladed beta-propeller, TolB-like | 1.42 |
